# Multivariate approach to identify electrophysiological markers for diagnosis and prognosis of schizophrenia

**DOI:** 10.1192/j.eurpsy.2021.1425

**Published:** 2021-08-13

**Authors:** L. Giuliani, N. Koutsouleris, T. Koenig, A. Mucci, A. Vignapiano, A. Reuf, M. Altamura, A. Bellomo, R. Brugnoli, G. Corrivetti, G. Di Lorenzo, P. Girardi, P. Monteleone, S. Galderisi, M. Maj

**Affiliations:** 1 Psychiatry, Università degli Studi della Campania “Luigi Vanvitelli”, Napoli, Italy; 2 Department Of Psychiatry And Psychotherapy, LMU München, München, Germany; 3 Psychiatry, University Hospital of Psychiatry, Bern, Bern, Switzerland; 4 Department Of Psychiatry, Univeristy of Campania Luigi Vanvitelli, Naples, Italy; 5 Psichiatria, ASL Napoli 1 - Centro, Napoli, Italy; 6 Psychiatry, University of Foggia, Foggia, Italy; 7 Psychiatry, University of Rome “La Sapienza”, Rome, Italy; 8 Department Of Psychiatry, 10European Biomedical Research Institute of Salerno (EBRIS), salerno, Italy; 9 Psychiatry, University of Rome “Tor Vergata”, Rome, Italy; 10 Department Of Medicine, Surgery And Dentistry “scuola Medica Salernitana”, University of Salerno, Baronissi/Salerno, Italy; 11 Department Of Psyschiatry, University of Campania “Luigi Vanvitelli”, Naples, Italy

**Keywords:** schizophrénia, EEG, machine learning, negative symptoms

## Abstract

**Introduction:**

Different electrophysiological indices have been investigated to identify diagnostic and prognostic markers of schizophrenia (SCZ). However, these indices have limited use in clinical practice, since both specificity and association with illness outcome remain unclear. In recent years, machine learning techniques, through the combination of multidimensional data, have been used to better characterize SCZ and to predict illness course.

**Objectives:**

The aim of the present study is to identify multimodal electrophysiological biomarkers that could be used in clinical practice in order to improve precision in diagnosis and prognosis of SCZ.

**Methods:**

Illness-related and functioning-related variables were measured at baseline in 113 subjects with SCZ and 57 healthy controls (HC), and after four-year follow-up in 61 SCZ. EEGs were recorded at baseline in resting-state condition and during two auditory tasks (MMN-P3a and N100-P3b). Through a Linear Support Vector Machine, using EEG data as predictors, four models were generated in order to classify SCZ and HC. Then, we combined unimodal classifiers’ scores through a stacking procedure. Pearson’s correlations between classifiers score with illness-related and functioning-related variables, at baseline and follow-up, were performed.

**Results:**

Each EEG model produced significant classification (p < 0.05). Global classifier discriminated SCZ from HC with accuracy of 75.4% (p < 0.01). A significant correlation (r=0.40, p=0.002) between the global classifier scores with negative symptoms at follow-up was found. Within negative symptoms, blunted affect showed the strongest correlation.

**Conclusions:**

Abnormalities in electrophysiological indices might be considered trait markers of schizophrenia. Our results suggest that multimodal electrophysiological markers might have prognostic value for negative symptoms.

